# Cecal Perforation Following Intraperitoneal Abscess after Antitubercular Therapy: A Case Report

**DOI:** 10.31729/jnma.8042

**Published:** 2023-02-28

**Authors:** Parikshit Prasai, Anjali Joshi, Santosh Poudel, Sarjan K.C., Rabin Pahari

**Affiliations:** 1 Kathmandu Medical College and Teaching Hospital, Sinamangal, Kathmandu, Nepal; 2 Department of Surgery, Kathmandu Medical College and Teaching Hospital, Sinamangal, Kathmandu, Nepal

**Keywords:** *case reports*, *cecum*, *intestinal perforation*, *tuberculosis*

## Abstract

Abdominal tuberculosis is defined as infection of gastrointestinal tract, peritoneum, abdominal solid organs, and/or abdominal lymphatics constituting approximately 12% of extra-pulmonary tuberculosis cases. Intestinal perforation is an acute presentation of abdominal tuberculosis. Intestinal perforation can occur before or at the beginning of anti-tubercular therapy. It is considered to be a paradoxical reaction if it occurs during or after treatment. Intestinal perforation is uncommon but serious and life-threatening as complication-mortality rate secondary to perforation are estimated to be >30%. We present a case of an 18-year-old female who developed cecal perforation following an intraperitoneal abscess after completion of anti-tubercular therapy for intestinal tuberculosis. She was a known case of intestinal tuberculosis. She had undergone pigtail catheterisation for an intraperitoneal abscess and completed 18 months of anti-tubercular therapy after which she developed cecal perforation. A paradoxical response was observed following the completion of antitubercular therapy. Early diagnosis and treatment reduce the complications and mortality rates of cecal perforation due to abdominal tuberculosis.

## INTRODUCTION

Abdominal tuberculosis (TB) is defined as infection of gastrointestinal tract, peritoneum, abdominal solid organs, and/or abdominal lymphatics. Abdominal TB constitutes approximately 12% of extra-pulmonary TB cases and 1 to 3% of total TB cases.^1,2^ Abdominal TB has acute presentations (e.g. perforation and obstruction) and a variety of chronic problems (e.g. vague ill health, anorexia, weight loss, malabsorption syndrome, subacute intestinal obstruction).^3,4^ The average time from the initial symptoms to perforation is 9 months and it can occur either before or at the beginning of TB treatment.^5^ Intestinal perforation occurring during or after treatment is suspected to be a paradoxical reaction.^5^ Intestinal perforation is uncommon but serious and life-threatening as complication-mortality rate secondary to perforation are estimated to be >30%.^6^ Here, we present a case of an 18-year-old female who developed cecal perforation following intraperitoneal abscess after completion of anti-tubercular therapy for abdominal TB.

## CASE REPORT

**Figure 1:**
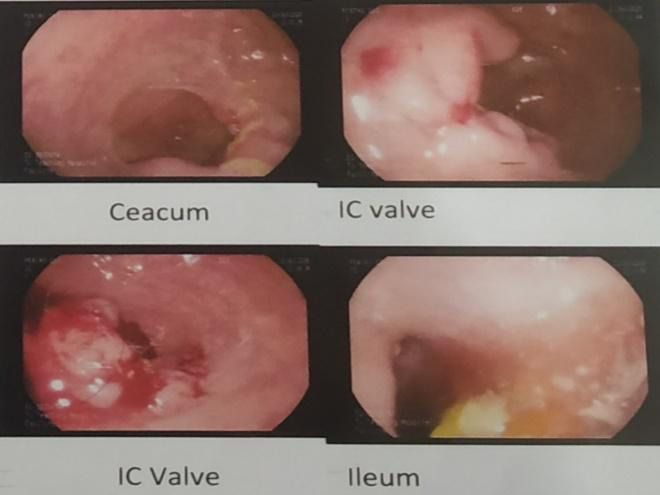
Nodules and transverse ulcer in terminal ileal and cecum.

An 18-year-old young lady was admitted to the emergency department of our institution with the chief complaint of localized abdominal pain over the right lower quadrant for 1 day which was non-radiating and aggravated on movement. She also complained of 5-6 episodes of vomiting for 1 day which was watery, nonblood stained and non-bile stained. On further inquiry, she gave a history of two episodes of loose stool for 1 day. The stool did not contain blood and mucus. She has a past history of primary ileocecal tuberculosis diagnosed 2 years ago in another tertiary centre. Colonoscopy done at that centre showed nodules and transverse ulcer in the terminal ileal and cecal region (Figure 1).

**Figure 2:**
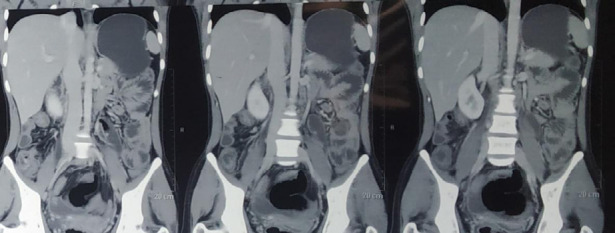
Well-defined cystic lesion seen within the pelvic cavity most likely representing intraperitoneal abscess.

Biopsy was taken from ileocecal valve ulcer and sent for histopathological examination (HPE). HPE showed granulomatous ileocolitis with an ulcer suggesting TB. So with a diagnosis of abdominal tuberculosis, ATT was started. After 7 months of initiation of the therapy, she presented to one of the tertiary centres with the chief complaint of pain in the right lower quadrant for 1 day. On contrast-enhanced Computed Tomography (CECT) abdomen and pelvis, a well-defined cystic lesion was seen within the pelvic cavity suggestive of an intraperitoneal abscess for which she underwent ultrasonography-guided pigtail catheterization (Figure 2). The catheter was placed for 7 days. She also completed 18 months of ATT.

On examination, she was hypotensive with BP 100/60 mm of Hg during the time of admission in our institution. On examination, there was localized tenderness over the right iliac fossa.

**Figure 3:**
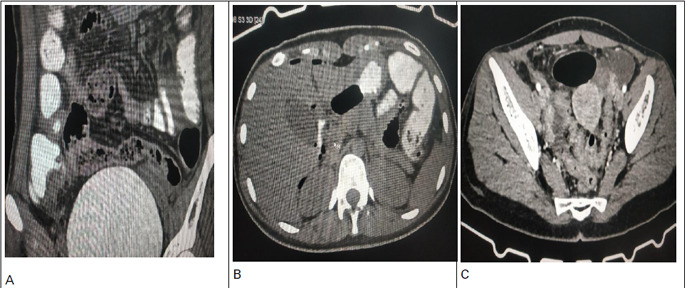
A) CT scan abdomen and pelvis showed thickening with ulcerated areas and subtle wall defect in the medial wall of the cecum with oral contrast extravasation, B) Pneumoperitoneum and C) Mild to moderate peritoneal collection.

Since her history and clinical findings were very suspicious of abdominal TB, CECT abdomen and pelvis were done along with other routine laboratory tests. Total leucocyte count was raised to 11,300/pl but values of the differential count were within the normal limit. CT scan abdomen and pelvis showed thickening with ulcerated areas and subtle wall defect in the medial wall of the cecum with oral contrast extravasation (Figure 3A) pneumoperitoneum (Figure 3B) and mild to moderate peritoneal collection (Figure 3C). All these findings on the CT abdomen was suggestive of cecal perforation.

Her vitals were monitored regularly. Exploratory laparotomy with right limited hemicolectomy with ileocolic side-to-side isoperistaltic anastomosis was planned. Perforation of 0.5 cm was present in the anterior wall of the cecum. Adhesion between the cecum, sigmoid colon, omentum and ileum was present. Around 10 cm of distal ileum wall thickening and stricture at the distal jejunum were noted.

Postoperatively, she was observed in the postoperative ward for 1 day and shifted to the surgery high care ward for 2 days and then shifted to the surgery ward for 2 days after her general condition improved. She was discharged on the 5^th^ post-operative day with analgesics and antibiotics and was advised for follow-up in outpatient department after 7 days. Her histopathological report was traced which showed lymphoid hyperplasia and transmural inflammation of the ileum without evidence suggestive of tuberculosis.

## DISCUSSION

Intestinal perforation occurs before or shortly after initiation of ATT, suggesting a natural progression of the condition.^7^ There have also been cases of intestinal perforation occurring after completion of ATT which has been hypothesized that a paradoxical response to treatment may be responsible for these cases.^7^ This paradoxical response is reported in about 2% of cases In the gastrointestinal tract and usually occurs between the first and sixth month of therapy.^5,8^ Similarly, intestinal perforation occurs between 3 weeks and 12 months after the initiation of ATT.^9^

In our study, symptoms of paradoxical response were reported after 7 months of initiation of ATT which was later diagnosed as an intraperitoneal abscess. Moreover, cecal perforation was reported after the completion of 18 months of ATT. Perforation is seen in male patients frequently.^9^ In contrast, in our case, the patient was female. Risk factors associated with paradoxical response include young age, high serum albumin, peripheral lymphadenopathies, absolute lymphocyte count less than 1000/mm^3^ and a haemoglobin concentration less than 10.5 g/dl.^10,11^ Our patient was also young. Factors associated with increased morbidity and mortality in this group of patients include delay in surgical treatment, presence of multiple perforations, primary closure of perforations, corticosteroid therapy, anastomotic leaks, advanced age and comorbidities.^8,9^ The best surgical approach for the management of TB-associated intestinal perforations is resection of the involved segment followed by an end-to-end anastomosis.^8,9^ In our case, exploratory laparotomy with right limited hemicolectomy with ileocolic side-to-side isoperistaltic anastomosis was performed.

Isolated cecal perforation, that too tubercular in origin, is a rare entity.^12^ Cecal perforations are commonly encountered as a part and parcel of various associated disease processes, in accordance with Laplace's law.^12^ Cecal perforations are usually seen associated with entities such as diverticular disease, inflammatory bowel diseases, Ogilvie's syndrome,^13^ closed loop obstructions,^14^ pancreatic carcinomas,^15^ colorectal cancers,^16^ Hirschsprung's disease,^17^ etc., in the presence of patent ileocecal valve. Furthermore, the exact mechanism responsible for an isolated involvement of the caecum remains unknown.

It is, therefore, necessary to think of a paradoxical response when a TB patient during or after antitubercular therapy develops a reappearance of signs and symptoms. Likewise, prompt diagnosis and surgery is required to prevent morbidity and mortality that arise due to life-threatening complications of paradoxical response such as intraperitoneal abscess and intestinal perforation. Information should be gathered from multiple studies to find the exact mechanism responsible for the isolated involvement of the cecum.
